# Dispersal and Repulsion of Entomopathogenic Nematodes to Prenol

**DOI:** 10.3390/biology8030058

**Published:** 2019-08-02

**Authors:** Kassandra Kin, Tiffany Baiocchi, Adler R. Dillman

**Affiliations:** Department of Nematology, University of California Riverside, Riverside, CA 92521, USA

**Keywords:** *Steinernema*, dispersal, emergence, prenol, chemotaxis

## Abstract

Chemosensory cues are crucial for entomopathogenic nematodes (EPNs)—a guild of insect-killing parasitic nematodes that are used as biological control agents against a variety of agricultural pests. Dispersal is an essential element of the EPN life cycle in which newly developed infective juveniles (IJs) emerge and migrate away from a resource-depleted insect cadaver in order to search for new hosts. Emergence and dispersal are complex processes that involve biotic and abiotic factors, however, the elements that result in EPN dispersal behaviors have not been well-studied. Prenol is a simple isoprenoid and a natural alcohol found in association with EPN-infected, resource-depleted insect cadavers, and this odorant has been speculated to play a role in dispersal behavior in EPNs. This hypothesis was tested by evaluating the behavioral responses of five different species of EPNs to prenol both as a distal-chemotactic cue and as a dispersal cue. The results indicate that prenol acted as a repulsive agent for all five species tested, while only two species responded to prenol as a dispersal cue.

## 1. Introduction

Entomopathogenic nematodes (EPNs) are insect-parasitic nematodes used as biological control agents against a variety of pests [1]. As part of the EPN life cycle, infective juveniles seek out and infect adults before developing into adults within the confines of their insect host. When resources are depleted from the cadaver, lack of food, along with other cues (including small molecules and pheromones) trigger the development of a modified third-stage juvenile–the infective juvenile (IJ) stage [2,3,4,5]—which disperses away from the depleted cadaver to search for a new host to colonize, in which it will resume development. These IJs are developmentally arrested, non-feeding, and are more tolerant to environmental stresses [5,6,7]. The IJs are the only stage which undergoes the single-time events of emergence and dispersal from the depleted cadaver to find, invade, kill and colonize a new insect host to continue their life cycle. These insect-killing capabilities are why EPNs are used in agriculture as a method of biological pest control [1] and as a model system to study host-parasite interactions.

The events of emergence and subsequent dispersal from the natal cadaver not only serve to help EPNs avoid or limit kin competition and inbreeding, but also to drive the newly-developed EPN IJs into the external environment where they will locate new suitable hosts in which they can resume their development [8,9,10]. Ascarosides (nematode pheromones) have been shown to stimulate dispersal behavior in nematodes, triggering juveniles to move quickly out of a central location [11]. However, EPN dispersal and the role of non-pheromone small molecules remains understudied.

Prenol (3-methyl-2-buten-1-ol) is an odorant associated with EPN-infected *Galleria mellonella* (Linnaeus, 1758) and has been shown to elicit repulsion from two EPN species: *Steinernema glaseri* (Steiner, 1929) and *Steinernema riobrave* (Cabanillas, Poinar and Raulston, 1994) [12]. Our study built upon this research, investigating additional EPN species, including *Steinernema feltiae* (Filipjev, 1934), *Steinernema carpocapsae* (Weiser, 1955), and *Heterorhabditis indica* (Poinar, Karunakar, David & David 1992). Moreover, this study focused on investigating the effects of post-collection age on both chemotaxis responses and dispersal responses to prenol in an effort to differentiate between these behaviors.

Despite the differences between dispersal and repulsion behavior, the distinction between these behaviors in EPNs remains unclear. Dispersal and chemotaxis are both natural events and appear to have some behavioral overlap but are set apart by context. As the insect cadaver becomes depleted of resources, a variety of environmental cues, including the absence of food, trigger IJ development [2,3,4,5]. Under these conditions, there is usually a high density of nematodes within the cadaver and a variety of odors and soluble chemicals—including pheromones—that might drive emergence and dispersal, which occur under specific circumstances. Chemotaxis, on the other hand, is a more general term referring to an organism’s response to chemical-based stimuli such as odors or soluble compounds, and is akin to other kinds of taxis such as responses to temperature (thermotaxis), or even responses to magnetic fields (magnetotaxis) [13,14,15]. Although dispersal behavior is a type of chemotaxis, we proposed that dispersal is a specific response that occurs in a specific context such as emergence from the cadaver, which may be influenced by various pressures and previous experiences (such as exposure to the odors or soluble compounds found in the natal cadaver). In this study, the term “chemotaxis” refers to the nematodes’ response to distal chemotactic cues, such as would occur during host-seeking by IJs. In this research, we addressed the effect of time post collection (after the IJs were removed from exposure to the cadaver’s odors and soluble chemicals) on the IJ response to prenol—as both a distal-chemotactic cue and a dispersal (direct-contact) cue.

## 2. Materials and Methods

### 2.1. Nematode Culturing

The following strains were cultured and used for experiments: *S. carpocapsae* (All), *Steinernema feltiae* (Strain SN), *Steinernema glaseri* (Strain NC), *Steinernema riobrave* (strain TX-355), and *Heterorhabditis indica* (Strain Hom-1) [16,17].

To culture the nematode strains used in the experiments, standard procedures were followed as previously described [13,18,19]. A 6-cm petri dish was lined with a 5.5-cm filter paper (Fisher Scientific: part number 09-795A; Fisherbrand, Pittsburgh, PA, USA). Six last instar *Galleria mellonella* larvae (waxworms) were then added to the plate along with approximately 30–50 nematodes per host to undergo the infection. Waxworms were purchased from CritterGrub (Wausau, WI).

The infections were then stored at room temperature (23–25 °C) for seven days (all species except *H. indica* which usually required 9–11 days of incubation to get a sufficient amount of IJs) and subsequently white-trapped. Infected waxworms were placed on white traps. The lid of a 35-mm petri dish was placed in the center of a 10-cm petri dish and was topped with a 7-cm filter paper which was lightly wetted with tap water. The infected and incubated waxworms were then placed on top of the filter paper and arranged in a star-like pattern to provide the newly emerged IJs sufficient room to emerge from the cadaver (only 5 to 6 wax worms were placed on each trap to avoid overcrowding). An amount of 6–7 mL of tap water was then added to the bottom of the plate to keep the filter paper wet and provide a reservoir for IJs to swim in until collection. Seven days after the infected worms were placed on a white trap, IJs were collected into 15-mL conical tubes. IJs that were to be used in behavioral assays were then rinsed three times in tap water before being stored in VWR 25-mL tissue culture flask at room temperature in clean tap water for no more than seven days per the requirements of the study. The densities of the IJ cultures varied between species: for *S. glaseri*, which is larger than the other species of nematodes [20], the density was kept below 3000 IJs/mL, and most cultures of *S. glaseri* were kept at approximately 2000 IJs/mL. For all the other species, the densities were kept between 2000 and 5000 IJs/mL, with most cultures having a density of around 3000 IJs/mL. The IJs used for emergence assays were not cleaned as described above in order to prevent the unnecessary loss of IJs, since they were to be quantified but not used for any further behavioral experiments. IJs were kept in the 15 mL conical vials until they could be counted (within seven days).

In future experiments, it may be advantageous to use a narrower window of time for IJ collection from white traps. In these experiments, we allowed IJs to emerge for seven days before collecting all of the IJs in a given white trap, however, emerging IJs could be collected every two days, providing a narrower age range in subsequent behavioral experiments.

### 2.2. Chemicals and Their Storage

Commonly known as prenol, 3-methyl-2-buten-1-ol, was obtained from Acros Organics (>99% pure prenol; part number: AC171920050). Due to the volatile nature of prenol, prenol was remade every two weeks and stored in amber glass vials. All aliquots of prenol were stored at room temperature with limited light exposure. For preparation and use of 2 M prenol (in experiments or in preparing further dilutions), adequate mixing should be carried out in order to ensure that the mixture is homogenous (since the solubility of prenol maxes out at approximately 2 M at room temperature). All preparations and dilutions of prenol were conducted using sterile MilliQ water.

### 2.3. Chemotaxis Assay

**Figure 1:**
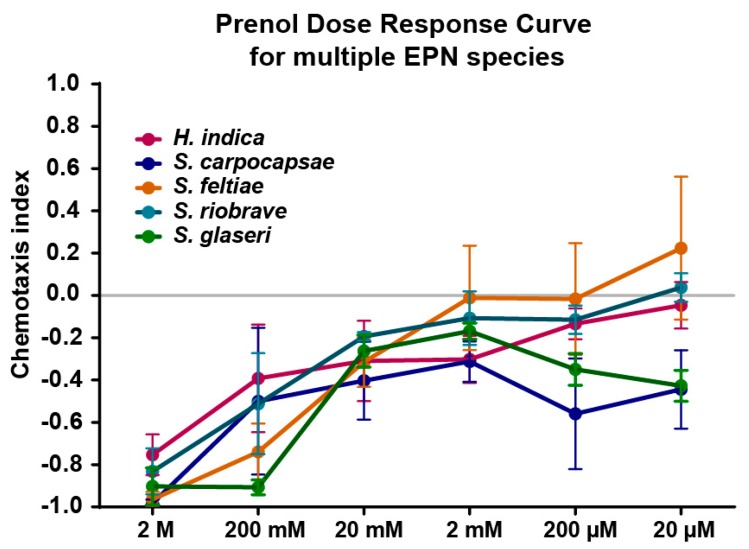
Dose response curve results for multiple EPN species (4-h post-collection IJs). Results of a standard chemotaxis assay where a chemotaxis index (y-axis) near +1 indicates high attraction and a score near −1 indicates high repulsion, with a score near 0 indicating no preference. The concentrations of prenol along the x-axis refer to the concertation that was put on the plate (agar). All the species were strongly repelled by 2 M and by 200 mM, while at 20 mM, repulsion was mitigated for most species (ranging between −0.2 and −0.45). Below 20 mM prenol, the responses varied from species to species, between a mostly neutral response to a slightly repelled response. Mean is shown and error bars represent SEM.

**Figure 2:**
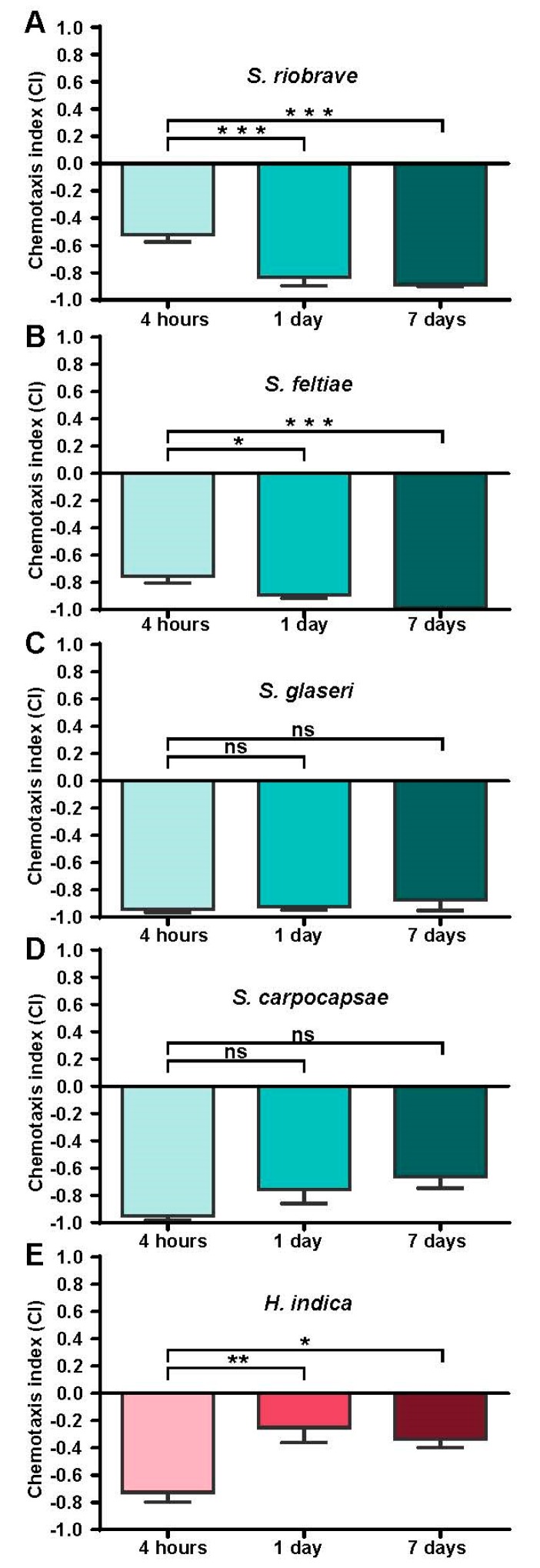
Chemotaxis index results for IJs at several times post collection (in response to 200 mM prenol). For chemotaxis index (CI) values: a score near −1 indicates high repulsion, near 0 indicates neutrality, and +1 indicates strong attraction. Statistical comparisons for chemotaxis indices were carried out using ordinary one-way ANOVA, stars indicate the results of the Tukey multiple comparisons test, indicating if the time post-collection had an effect on the response of EPN to prenol as a distal-chemotactic cue. Mean is shown and error bars represent SEM. * *p* < 0.05; ** *p* < 0.01; *** *p* < 0.001.

Chemotaxis assays were conducted as previously described [2,3,4] (Chemotaxis assays used to collect data displayed in Figure 1 and Figure 2). A template for scoring (Figure S1 was placed on the bottom of a 10-cm chemotaxis plate, chemotaxis media was made as previously described [21]. To each plate, 2 µL of 1 M sodium azide (a paralytic) was placed on each scoring circle. An amount of 5 µL of 200 mM prenol, diluted in sterile Milli Q water, was then applied to one of the scoring circles, and on the opposing scoring circle, 5 µL of sterile Milli Q water (serving as the control) was applied. IJs were then placed in the center of the chemotaxis plate. A 5 µL pellet containing approximately 250 IJs was placed in the center of the plate (the pellet was prepared by pipetting a calculated volume of IJs based on the density of the collection and IJs were allowed to settle to the bottom of the 1.5 mL tube before being pipetted as a pellet in approximately 5 µL). Each experimental condition consisted of three plates run simultaneously, stacked such that each plate was turned 120 degrees to account for side bias in the assay. The stack of three plates was placed in an empty freezer box and was left on an anti-vibration platform for the duration of approximately 1 h at room temperature (24 °C ± 1 °C). At the termination of the assay, the plates were scored using the following method. The IJs in the scoring circles were counted first, providing the chemotaxis index (a minimum of 7 IJs between the control and test circle were required in order for the data to be recorded, the same threshold as was used in a previous study [12]). The entire population of nematodes on the plate was then counted and scored based on their placement on the template in order to see the participation of the IJs (participation data is available in Supplementary Figure S2). For all species and conditions, 6 experiments were conducted. For each experiment, a total of 3 technical replicates (3 plates run in parallel) were completed, except for *H. indica* experiments, where 9 plates were run to help account for observed higher variability in behaviors and to assist in our initial investigation of this particular genus’ behavior in response to prenol.

### 2.4. Dose Response Curve Experiments

For the dose response curve, the IJs were cultured as described above. Within 4 h of being collected from the white trap, IJs were placed on a chemotaxis plate with a template attached and the methods described in *Chemotaxis Assay* were used to conduct these assays. Three experiments consisting of 3 plates each were ran for each of the *Steinernema* species. For, *H. indica*, there was a slightly higher variability in the results, thus, a fourth experiment was completed.

### 2.5. Timing Assay

**Figure 3:**
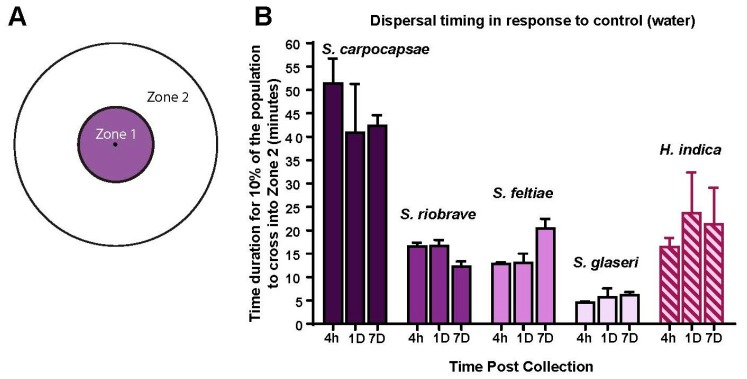
Timing assay information. (**A**) Template for timing and dispersal assays, the black dot in the center of zone 1 represents the initial placement of nematodes. (**B**) Results of timing assays for each species and time post-collection. Along the X-axis the time post-collection is listed: 4 h, 1 day and 7 days. Evaluations for each species and time point post-collection were conducted with photography (see methods) to determine how long it took for the IJs to cross completely over into zone 2 from zone 1 (shown in panel A). Mean is shown and error bars represent SEM; statistical analysis used Repeated Measure (RM) *two-way ANOVA*, and *Tukey’s multiple comparisons test*, but no statistical differences were found within each species dispersal across the time post-collection.

Timing assays were conducted in order to determine the duration of the dispersal assays. A template (Figure 3B) was placed on the bottom of a chemotaxis plate, and the experimental design was modified after Kaplan et al., 2012, while the template design was from Castelletto et al., 2014. The chemotaxis plate media was made in the same fashion as the plates used in the chemotaxis assays [21]. The template design consisted of two concentric circles (the outer one fit just within the rim of the 10 cm petri dish bottom). The inner circle had a diameter of 3 cm, meaning that IJs would have to travel approximately 1.5 cm to move outside of the inner zone (denoted as zone 1). Cross-sectional lines were used to assist in counting.

A pellet of 250 IJs was placed in the center of a chemotaxis plate. The excess water from the pellet was left to dry and once it had, 2 µL of Milli Q water was placed directly upon the nematodes. The plate was then photographed with a camera (Cannon EOS Rebel T5i EOS 700D with attached Macro lens—Cannon EF 100 mm f/2.8L Macro IS USM) and adjusted for clarity. As soon as the first nematodes broke the surface tension of the water, the time-lapse photos began. The photo periods for each species were as follows: 30-s intervals were allotted for *S. glaseri*, *S. riobrave*, and *S. feltiae*. *S. carpocapsae*, due to its known ambusher foraging strategy, was photographed every 60 s. The assays lasted a total of 20 min for *S. glaseri*, 30 min for *S. feltiae* and *S. riobrave*, and 60 min for *S. carpocapsae.* All assays were performed at room temperature (24 °C ± 1 °C) with ambient overhead lighting (from fluorescent tube lighting diffused by plastic covers located approximately 200 cm above the assay plate). This lighting was sufficient for obtaining the photos without exposing the nematodes to excess light or heat from light bulbs. Immediately after the last photo was taken, the plates were counted to determine the total number of nematodes on the plate. Image sequences were then analyzed to determine when 9–15% of the population had fully crossed the boundary between zone 1 and zone 2. Timing was recorded and used to establish the duration of the dispersal assays for each species. We would like to note that *S. glaseri* dispersed quickly in our control (Milli Q water), thus, we opted for a slightly longer dispersal assay duration, which aligned with 9–13% of the population crossing the border. This was carried out for logistical purposes, as it was far easier to run a 9-min assay than a 5-min assay. To time assays, each experimental condition (each time point, for each species) consisted of a total of 3 experiments and each experiment consisted of 3 sequential runs (run one after another, since only one camera was available).

### 2.6. Dispersal Assay

In order to determine the dispersal behavior of the IJs when in contact with prenol, a dispersal template (Figure 3B) was added to the bottom of a chemotaxis plate. A nematode pellet containing approximately 250 IJs was placed in the center of the plate. The pellet was allowed to dry, and then either 2 µL of water (which served as our baseline and control) or of prenol (200 mM) was applied to the nematodes. Once the applied liquid had soaked into the agar and the nematodes had broken the surface tension, the plates were stored in freezer boxes and placed onto anti-vibration platforms (at room temperature (24 °C ± 1 °C)) for the duration of the assay. The length of the assay varied based on the nematode species. A total of 9 min was allotted for *S. glaseri*, 14 min to *S. riobrave*, and *S. feltiae*, 20 min was allotted for *H. indica*, and *S. carpocapsae* was given 55 min to disperse, as determined via the timing assays. The plates were prepared individually with approximately 5-min time gaps in between each plate to allow enough time for quantification. At the termination of the assay, the nematodes were scored. The nematodes that had fully crossed outside of zone 1 and were no longer touching the border were counted first and were classified as being “dispersed”. The nematodes that were touching the border line were counted and considered to have not fully crossed into zone two of the template. Lastly, the nematodes within the center of the circle were counted and since they remained in the center of the plate, they were classified as not exhibiting dispersal behavior. Each experimental condition (each time point, for each species) consisted of four experiments, each conducted with 3 technical replicates (3 plates run in parallel).

For the chemotaxis, timing, and dispersal assays, IJs that had been collected within 4 hours, 1 day or 7 days prior to the experiment were used. These timepoints were selected for logistical purposes (as well as potentially ecologically-relevant changes that might occur with time post-removal/isolation away from the cadaver) given the time frames of infection incubations, white trap incubations and holding time before the experiment.

For both the chemotaxis and dispersal experiments, separate batches of nematodes were used for each replicate (i.e., each 4-h experiment was conducted using a batch from a separate infection white-trap batch). For each experiment across the time points, we used the batches consistently (i.e., the batch used for our first 4-h experiment, the first 24-h experiment and the first 7-day experiment all came from the same batch of collected nematodes).

### 2.7. Statistical Analysis

All statistical analyses were conducted using GraphPad Prism software. Analysis of chemotaxis indices utilized an ordinary *one-way ANOVA* and *Tukey’s multiple comparisons test*, comparing the means of every column to every other column. For participation values (from chemotaxis assays), a regular *two-way ANOVA* was utilized with *Tukey’s multiple comparisons test*. Analyses compared only within the *test*, *middle*, and *control* sides but not between these regions.

For the timing assays, we used a Repeated Measures (RM) *two-way ANOVA* with *Tukey’s multiple comparisons test* (comparing the behaviors within each species). Additionally, another RM *two-way ANOVA* with *Tukey’s multiple comparison test* was run to compare behaviors between species (i.e., comparing the behaviors of species to one another, rather than the time-post-collection age within a single species).

Dispersal assays involved an RM *two-way ANOVA* with *Sidack’s multiple comparisons test* to evaluate shifts in behavior within each species and between the time points post collection.

## 3. Results

### 3.1. Repulsion to Prenol is Conserved Among EPNs 

We evaluated five species of EPNs, *Steinernema carpocapsae*, *S. feltiae*, *S. glaseri*, *S. riobrave*, and *Heterorhabditis indica*, two of which had previously been shown to be repelled by prenol at varying doses (*S. glaseri* and *S. riobrave*) [12]. The results revealed that EPNs were strongly repelled by doses of 200 mM or higher, and that species responses were comparable for most doses (Figure 1).

The responses of the five species were also evaluated at varying ages post collection, at 4 h, 1 day, and 7 days (post collection) (Figure 2). The results showed that two of the four species (*S. riobrave* and *S. feltiae*) exhibited significantly higher repulsion from prenol (200 mM) at 1 day and 7 days compared to the 4-h post-collection time point (Figure 2A,B). However, *S. glaseri* and *S. carpocapsae* did not exhibit any significant behavioral shifts in relation to their post-collection age (Figure 2C,D). *H. indica* exhibited a unique response among the EPNs tested in that repulsion was significantly reduced after 24 h from collection (Figure 2E).

### 3.2. Dispersal Speed Is Consistent across Time Post-Collection

We evaluated the effect of post-collection age on motility using a 2-zone dispersal assay which we constructed based on previous work [11,22] (Figure 3A). The data revealed that post-collection age had no significant effects on the motility of each species (Figure 3B) and also provided information for approximately how long the dispersal assay should be for each species to allow for approximately 10% of the population to cross the boundary of zone 1 into zone 2. These experiments also yielded information on appropriate timing measures to be used in the following dispersal assays: 60 min for *S. carpocapsae*, 14 min for *S. riobrave* and *S. feltiae*, 9 min for *S. glaseri* (for logistical reasons, a slightly longer amount of time was chosen) and 20 min for *H. indica*.

### 3.3. Prenol Acts as a Dispersal Cue for Some EPNs

**Figure 4:**
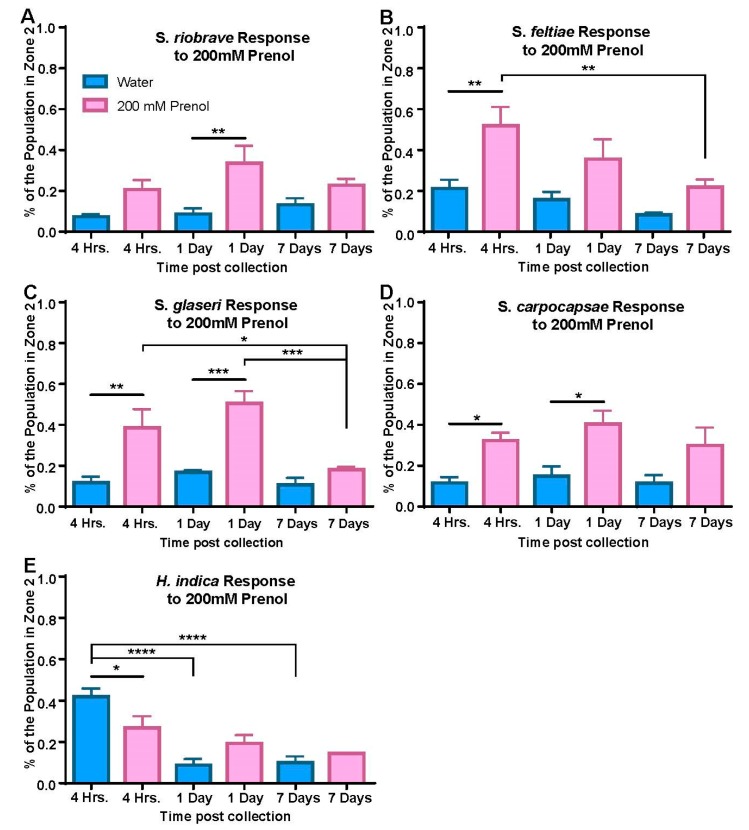
Dispersal assay results for different species of EPNs. Responses to water are shown in blue and responses to prenol (200 mM) are shown in pink. Assays were run for a different length of time for each species (*S. riobrave*, *S. feltiae*, and *H. indica* were run for 15 min, *S. glaseri* was run for 10 min and *S. carpocapsae* was run for 1 h). After the elapsed time, assays were scored to evaluate what percentage of the population had moved completely into zone 2 (see Figure 3A for breakdown of scoring regions). Statistical analysis used two methods: the first was a comparison between water and prenol, done using an RM *two-way ANOVA* with *Sidack’s multiple comparisons test*, results shown as a simple bar above prenol and water. A second statistical analysis (using the same parameters as listed above) was used to compare the general responses to prenol over the different time points post-collection.

To evaluate the effects of prenol (200 mM) on dispersal behavior among the five species, we used the dispersal assay template previously used for evaluating motility and timing the IJs (Figure 3A). Both prenol and a control (water) were evaluated for how they affected dispersal from the center of the arena (Figure 4A–E). It was our hypothesis that younger IJs (4 h–1 day post-collection) would experience significantly higher dispersal compared to older (7-day post-collection) IJ populations (see Figure S3). Such a trend was only seen in two of the five species: *S. feltiae* and *S. glaseri* (Figure 4B,C). Although *S. carpocapsae* and *S. riobrave* (Figure 4A,D) had significantly higher dispersal compared to water, the trends did not reflect what we would expect to be indicative of response to a dispersal cue (with diminished respond to prenol in relation to the post-collection age). Interestingly *H. indica* (Figure 4E) did not show a strong response to prenol at any of the post-collection time points; instead, water appeared to elicit an increase in dispersal compared to prenol at the earliest post-collection time (4 h). Additionally, IJs exhibited significantly lower dispersal (to water) by 1 day post-collection.

## 4. Discussion

Although much research has been conducted to evaluate chemotactic responses of EPNs to odors or different sources of odorants in the context of host-seeking [13,15,18,22,23,24,25], EPN dispersal in response to chemical stimuli has remained relatively understudied, with previous research focusing primarily on ascarosides (nematode pheromones) [11]. The odor prenol has been found in association with *S. glaseri*- and *S. riobrave*-infected cadavers, and it was previously hypothesized that prenol might serve as a dispersal cue [12]. The work reported here has built upon these findings, revealing that other species of steinernematids respond to prenol in the same way as was reported for *S. gaseri* and *S. riobrave* [12], and that distantly related *Heterorhabditis indica* was also repelled by prenol. We did note that in the dose response cuves for *S. glaseri*, the IJs appeared to be slightly more repelled than previously reported. This may be due to normal variations in behavior or perhaps to the use of a very narrow post-collection age (4 h) in this study, as opposed to the wide post-collection age in the Baiocchi et al., 2017 study (which used IJs between the initial harvest from white traps all the way out to 14 days post-collection). In contrast to the previous study, the current research primarily focused on the aspect of post-collection age within a specific time frame, allowing us to more closely evaluate behavioral shifts that occur as a result of time spent away from the natal cadaver (post-collection age), which may also represent time away from pheromones and other molecules such as waste- or decay-related products that may be in relatively high concentrations in a resource-depleted and overcrowded cadavers. Furthermore, our use of dispersal assays was aimed at evaluating each species’ response to prenol as a potential dispersal cue, as had been speculated in previous work [12].

Although age is implicated in a variety of behavioral shifts, we observed that general motility was not significantly affected. However, it was clear that motility between species was a major factor—especially in the case of *S. carpocapsae*, a known ambusher [26]. Since each species was given an appropriate amount of time for the dispersal assay and motility across the post-collection ages did not shift significantly for any, we propose that the shifts in behavior observed in the dispersal assay were most likely caused by changes in sensitivity to prenol, indicating the use of prenol as a dispersal cue. We hypothesized that younger IJs (either 4 h or 1 day post-collection) would exhibit significantly higher dispersal than older IJs (as is depicted in Figure S3). This trend was only seen for two of the tested species: *S. glaseri* and *S. feltiae*. Moreover, the behavioral trends exhibited in the dispersal assays by these two species are not simply a reflection of the CI values displayed in Figure 2. Whereas *S. glaseri* repulsion did not appear to significantly change with respect to post-collection age, the response to prenol as a dispersal cue dropped drastically by 7 days post-collection. Perhaps even more compellingly, *S. feltiae* IJs exhibited stronger repulsion with increased time post-collection, whereas in the dispersal assays, the opposite trend was seen, where the IJs responded most strongly at the 4-h time point and dispersal dropped significantly by 7 days post-collection. These two cases indicate that although prenol may be a repulsive chemotactic cue, the dispersal assay measures dispersal, not chemotaxis, and that it appropriately serves to evaluate dispersal as a different facet of EPN behavior.

## 5. Conclusions

The current research demonstrated that post-collection age impacts behavioral responses to prenol for some EPN species (*S. feltiae*, *S. riobrave*, and *H. indica*). Prenol may also serve as one of the many cues that plays a role in IJ dispersal of the EPN species *S. glaseri* and *S. feltiae*. Improving our understanding of EPN behaviors—such as dispersal behavior—will continue to require more research. However, this study provides an easy quantitative approach to evaluating dispersal behavior, which could potentially be used in future studies for evaluating other odors such as those associated with EPN infections or even ascarosides.

## Supplementary Materials

The following are available online at https://www.mdpi.com/2079-7737/8/3/58/s1, Figure S1: Scoring template for chemotaxis assays, Figure S2: Participation scores from chemotaxis assays in Figure 1. Figure S3: Graphical representation of our hypothesis or predicted behavioral trends. All relevant data is listed within the paper and within the supplementary materials, Raw data and other information are available from the corresponding author upon reasonable request.
