# Supplementary material for: Dispersal and Repulsion of Entomopathogenic Nematodes to Prenol
Source: Biology (Basel). 2019 Aug 2;8(3):58. doi: 10.3390/biology8030058 (PMC6783912; doi:10.3390/biology8030058)
Supplement: Supplementary file 1 [file biology-08-00058-s001.pdf]

biology

MDPI

Supplementary Figure legends

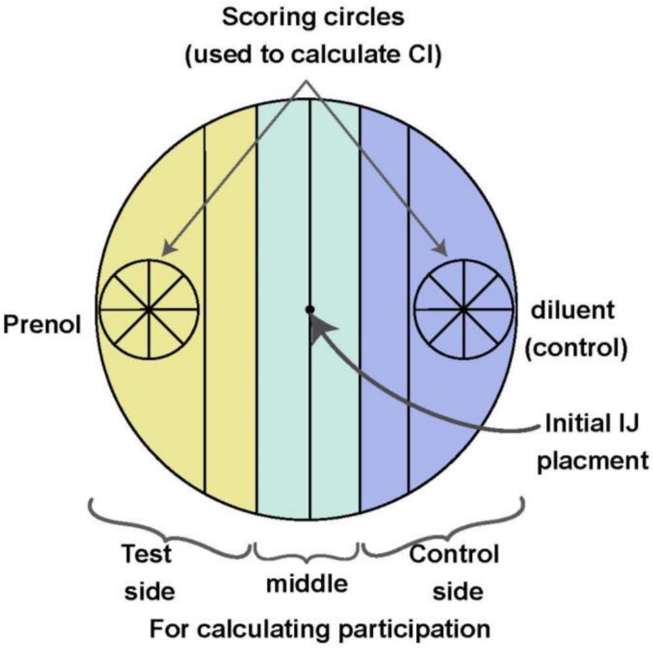

Figure S1. Scoring template for chemotaxis assays.

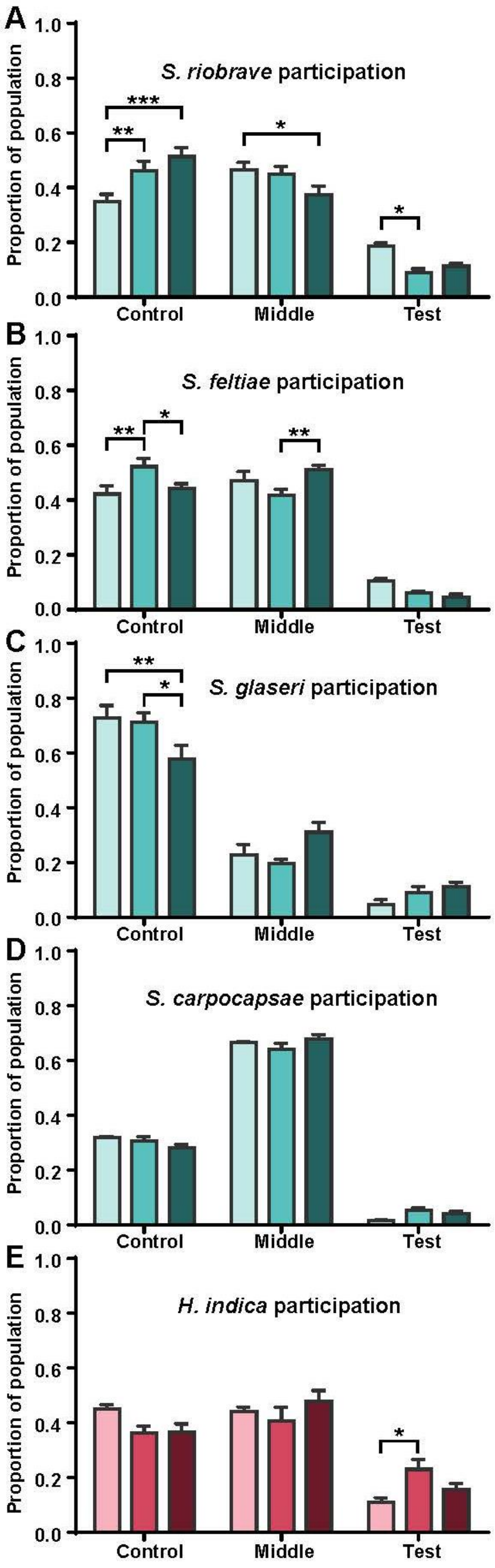

Figure S2. Participation scores from chemotaxis assays in Figure 1. Participation reflects that *S. riobrave S. feltiae* and *S. glaseri* and *H.* indica responded with high participation (above 50% of the population moving from the center region) while *S. carpocapsae* had very low participation (less than 50% moving out of the center region).

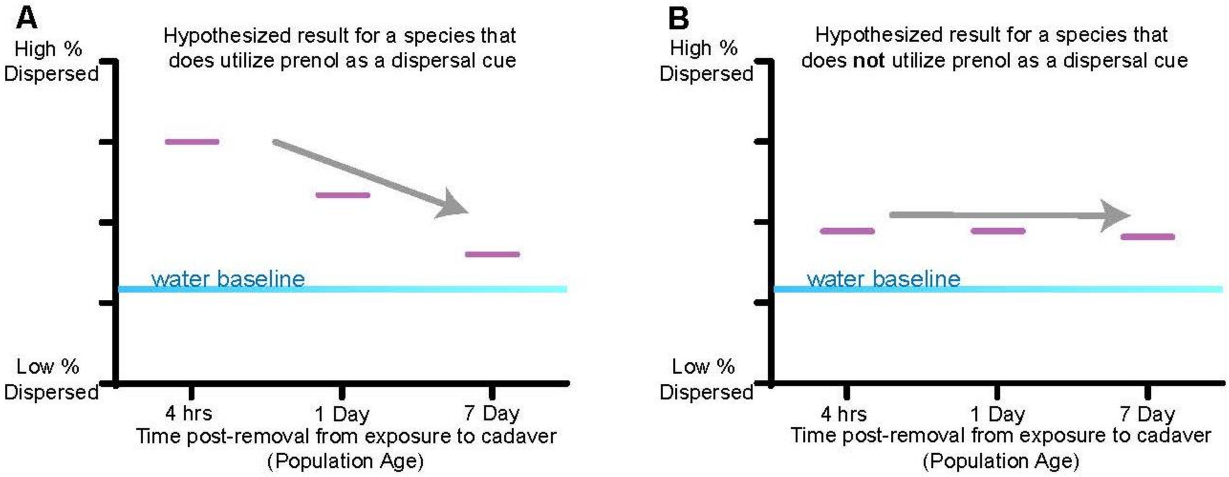

Figure S3. Graphical representation of our hypothesis or predicted behavioral trends for **A.** IJs that do respond to prenol as a dispersal cue **B.** IJs that do not respond to prenol as a dispersal cue.
